# TNF inhibits production of stromal cell-derived factor 1 by bone stromal cells and increases osteoclast precursor mobilization from bone marrow to peripheral blood

**DOI:** 10.1186/ar2391

**Published:** 2008-03-27

**Authors:** Qian Zhang, Ruolin Guo, Edward M Schwarz, Brendan F Boyce, Lianping Xing

**Affiliations:** 1 Department of Pathology and Laboratory Medicine, University of Rochester Medical Center, 601 Elmwood Avenue, Box 626, Rochester, NY 14642, USA; 2 Center for Musculoskeletal Research, University of Rochester Medical Center, Rochester, NY 14642, USA

## Abstract

**Introduction:**

The objective of the present study was to investigate the role of the stromal cell-derived factor 1 (SDF-1)/CXCR4 axis in TNF-induced mobilization of osteoclast precursors (OCPs) from bone marrow.

**Methods:**

OCPs were generated from bone marrow cells of TNF-transgenic mice or wild-type mice treated with TNF or PBS. The percentage of CD11b^+^/Gr-1^-/lo ^OCPs was assessed by fluorescence-activated cell sorting. OCP migration to the SDF-1 gradient and the osteoclast forming potency were assessed in chemotaxis/osteoclastogenic assays. SDF-1 expression was assessed by real-time RT-PCR, ELISA and immunostaining in primary bone marrow stromal cells, in the ST2 bone marrow stromal cell line, and in bones from TNF-injected mice.

**Results:**

OCPs generated *in vitro *from wild-type mice migrated to SDF-1 gradients and subsequently gave rise to osteoclasts in response to RANKL and macrophage colony-stimulating factor. TNF reduced SDF-1 expression by ST2 cells. Bone marrow stromal cells from TNF-transgenic mice produced low levels of SDF-1. TNF treatment of wild-type mice decreased the SDF-1 concentration in bone marrow extracts and decreased the SDF-1 immunostaining of bone marrow stromal cells, and it also increased the circulating OCP numbers. The percentage of bone marrow CXCR4^+ ^OCPs was similar in TNF-transgenic mice and wild-type littermates and in TNF-treated and PBS-treated wild-type mice.

**Conclusion:**

Systemically elevated TNF levels inhibit bone marrow stromal cell production of SDF-1 and increase the release of bone marrow OCPs to the peripheral blood. Disruption of the SDF-1/CXCR4 axis by TNF may play an important role in mediating OCP mobilization from the bone marrow cavity in chronic inflammatory arthritis.

## Introduction

TNF is a clinically validated etiological factor in inflammatory-erosive arthritis and is known to synergize with RANKL and macrophage colony-stimulating factor (M-CSF) to enhance the differentiation of osteoclast precursors (OCPs) into bone-resorbing osteoclasts in inflamed joints [1,2]. Patients with psoriatic arthritis [3] and mice with TNF-induced arthritis [4,5] have increased numbers of circulating OCPs, which correlate with systemically increased TNF concentrations and are reduced by anti-TNF therapy in association with clinical improvement. These findings suggest that OCP mobilization from the marrow may be involved in the pathogenesis of inflammatory arthritis. The factors that mediate OCP mobilization are currently unknown.

Stromal cell-derived factor 1 (SDF-1), a member of the C-X-C chemokine family also known as CXCL12, acts through its receptor CXCR4, and is the master chemokine that modulates trafficking of hematopoietic stem cells and progenitors [6,7]. Studies of knockout mice reveal that the SDF-1/CXCR4 axis is required for fetal B lymphopoiesis, bone marrow myelopoiesis and organogenesis [8-11]. Both SDF-1-deficient and CXCR4-deficient mice die perinatally and have very few hematopoietic stem cells and progenitors within their bone marrow. SDF-1 and CXCR4 have been implicated in OCP migration *in vitro*, and SDF-1 treatment of OCPs increases osteoclastogenesis and subsequent osteoclast bone-resorbing capacity [12,13].

SDF-1 is primarily produced by bone marrow stromal cells, such as osteoblasts and endothelial cells [14]. Expression of SDF-1 is controlled by various factors including hypoxia [15], DNA damage [14] and cytokines, such as transforming growth factor beta (TGFβ) [16] and granulocyte colony-stimulating factor (G-CSF) [17]. G-CSF is used clinically to stimulate the release of hematopoietic stem cells from the bone marrow into the bloodstream of patients with a variety of malignancies. The stem cells are then harvested from the blood as a source of stem cells to be returned to patients following chemotherapy or bone marrow transplantation. Whether or not inflammatory cytokines such as TNF affect the SDF-1/CXCR4 axis *in vivo *to control OCP mobilization, however, has not been studied.

We used TNF-transgenic (TNF-Tg) mice as a model of chronic TNF overexpression and also injected WT mice with TNF as an acute model to investigate the involvement of TNF in the SDF-1/CXCR4 axis control of OCP mobilization. We found that TNF directly inhibits SDF-1 production by bone marrow stromal cells and that it has little effect on CXCR4 expression by OCPs. A mechanism whereby TNF accelerates OCP mobilization in inflammatory erosive arthritis may therefore be to reduce bone marrow SDF-1 concentrations.

## Materials and methods

### Reagents and animals

Recombinant murine SDF-1, TNFα, and RANKL were from R&D Systems (Minneapolis, MN, USA). Allophycocyanin–anti-murine CD11b (M1/70) was from eBiosciences (San Diego, CA, USA). FITC–anti-murine Gr-1 (RB6-8c5), biotin–anti-CXCR4 (2B11/CXCR4) and streptavidin–PE-Texas Red conjugate were from BD PharMingen (San Diego, CA, USA). Mouse SDF-1/CXCL12 DuoSet Development system was from R&D Systems.

TNF-Tg mice in a CBA × C57BL/6 background (3647 TNF-Tg line) were obtained originally from Dr G. Kollias and were characterized by our group previously [4]. TNF-Tg mice have been bred with C57/B6 mice for eight generations. *Cxcr4 floxed *and *CD11b*^+^*/Cre *mice were obtained from Dr YR Zou [18] and Dr J Vacher [19], respectively. Both types of mice are in a C57BL/6 background.

TNF was given by subcutaneous injection, as described previously [4]. The University Committee on Animal Resources of the University of Rochester approved all studies.

### Chemotaxis/osteoclastogenesis assay

Freshly isolated bone marrow cells were cultured with M-CSF in α-modified essential medium (Invitrogen, San Francisco, CA, USA) supplemented with 10% fetal bovine serum (Invitrogen) for 3 days, and adherent cells were used as OCPs. Assays were performed using transwell chemotaxis inserts with 5-μm-pore polycarbonate filters (Corning Costar, Acton, MA, USA). OCPs were labeled with Calcein AM (Molecular Probes, Carlsbad, CA, USA) at a final concentration of 2 μg/ml, and 100 μl (10^6 ^cells) cell suspension were loaded into the upper chamber of a transwell insert. The transwell inserts were immediately moved to wells of a 24-well tissue culture dish containing different doses of SDF-1α (1, 10 or 100 ng/ml). After 3 hours of incubation, the migrated cells in the bottom wells were collected, centrifuged and solubilized (in 100 μl Hank's Buffered Salt Solution with 1% SDS/0.2 N NaOH). The calcein label was read in a 96-well FluoroNunc plate (Nalge Nunc International, Rochester, NY, USA) and quantified in a Gemini XS microplate spectrofluorometer (Molecular Devices, Sunnyvale, CA, USA) at 485 nm/530 nm.

The number of cells that migrated was calculated according to a standard curve generated by plotting the calcein intensity of serially diluted labeled cells versus the cell numbers. The percentage of migrated cells was calculated as follows: (migrated cell number/total loaded cell number) × 100%. The cells in the upper and lower chambers of the transwell were collected and cultured with M-CSF and RANKL to determine whether they could differentiate into osteoclasts, as described previously [4]. These treated cells were fixed and stained for tartrate-resistant acid phosphatase activity to identify osteoclasts. Tartrate-resistant acid phosphatase-positive cells containing ≥ 3 nuclei were counted as mature osteoclasts.

### Fluorescence-activated cell sorting analysis

Bone marrow cells or peripheral blood were freshly isolated, stained with various fluorescence-labeled antibodies, and subjected to fluorescence-activated cell sorting (FACS) analysis, as described previously [4,20].

### Quantitative real-time PCR

Total RNA was extracted using TRIzol reagent (Invitrogen, Carlsbad, CA, USA) and cDNA was synthesized by the RNA PCR Core Kit (Applied Biosystems, Branchburg, NJ, USA). Quantitative PCR amplification was performed with gene-specific primers using an iCycler iQ Multiple-Color Real-Time PCR Detection System (Bio-Rad Laboratories, Hercules, CA, USA), as described previously [20].

The primer sequences are as follows: *SDF-1*, forward 5'-GCTCTGCATCAGTGACGG TA-3' and reverse 5'-TAATTACGGGTCAATGCACA-3' ; *CXCR4*, forward 5'-CTTTGTCATCACACTCC-CCTT-3' and reverse 5'-GCCCACATAGACTGCCT-TTTC-3' ; *TGF-β*, forward 5'-TCACTGGAGTTGTACGGCAG-3' and reverse 5'-TCTCTGTGGAGCTGAAGCAA-3' ; *G-CSF*, forward 5'-GCTGCTGCTGT-GGCAAAGT-3' and reverse 5'-AGCCTGACAGTGACCAGG-3' ; and *actin*, forward 5'-ACCCAGATCATGTTTGAGAC-3' and reverse 5'-GTCAGGATCTTCATGA-GGTAGT-3'.

A relative standard curve method was used to calculate the amplification efficiency. The standard curve was made from six points corresponding to 10-fold cDNA dilution series. For each sample, the relative amount was calculated from its respective standard curve. Standards and samples were run in triplicate.

### Enzyme-linked immunosorbent assay

Culture supernatants were collected from primary stromal cells and from the ST2 stromal cells. ELISA was performed with the Mouse SDF-1/CXCL12 DuoSet Development system. Ninety-six-well EIA/RIA plates (Costar, Corning, NY, USA) were coated with a capturing monoclonal antibody to SDF-1 and were then blocked with a mixture of 1% bovine serum albumin, 0.05% NaN_3 _and 5% sucrose in PBS. Culture supernatants were diluted in reagent diluent (1% bovine serum albumin in PBS) and incubated for 2 hours at room temperature. The detection antibody was diluted in reagent diluent and incubated for 2 hours at room temperature. Antibody binding was detected with streptavidin-conjugated horseradish peroxidase and developed with a substrate solution (1:1 mixture of H_2_O_2 _and tetramethylbenzidine).

A standard curve was generated for each set of samples assayed and was made from seven points of a twofold dilution series. Each standard or sample was assayed in duplicate.

### Preparation of bone sections and immunohistochemistry

Long bones from mice treated with TNF or PBS were fixed in 10% phosphate-buffered formalin, decalcified in 10% ethylenediamine tetraacetic acid and embedded in paraffin wax. Deparaffinized sections were quenched with 3% hydrogen peroxide and were treated for antigen retrieval for 30 minutes. Sections were then stained with a rabbit anti-SDF-1 antibody (Santa Cruz Biotechnology, Santa Cruz, CA, USA) and immunostaining was performed.

### Generation of *Cxcr4f/f/CD11b*^+^/*Cre *conditional knockout mice

*Cxcr4 floxed *female mice were bred with *CD11b*^+^*/Cre *male mice to generate the *Cxcr4*^+^*/f/CD11b*^+^*/Cre *F1 generation. *Cxcr4*^+^*/f/CD11b*^+^*/Cre *male mice were then crossed with *Cxcr4f/f *female mice to produce *Cxcr4f/f/CD11b*^+^*/Cre *conditional knockout mice (CXCR4 CKO). Each litter comprised five to eight pups, indicating that deletion of CXCR4 in CD11b^+ ^cells does not cause embryonic death. CXCR4 CKO mice were identified by PCR genotyping. The efficiency of CXCR4 deletion in the bone marrow CD11b^+ ^cells was assessed by FACS analysis using FITC–anti-CD11b and allophycocyanin–anti-CXCR4 antibodies.

### Statistical analysis

All results are presented as the mean ± standard error of the mean. Comparisons were made by analysis of variance and Student's *t *test for unpaired data. *P *< 0.05 was considered to represent statistical significance.

## Results

### SDF-1 has a chemotaxic effect on bone marrow OCPs

We and other workers have demonstrated that patients or mice with chronic inflammatory arthritis have an increased frequency of OCPs in peripheral blood and spleens, and that TNF promotes the release of bone marrow OCPs into the bloodstream [3-5]. To investigate whether the SDF-1/CXCR4 axis – the master chemokine system controlling mobilization of hematopoietic stem cells and progenitors – mediates TNF-induced OCP mobilization, we first verified that OCPs express functional CXCR4 and migrate toward a SDF-1 gradient in a combined chemotaxis/osteoclastogenesis assay. M-CSF-dependent bone marrow mononuclear cells were generated *in vitro *and were used as the source of OCPs. To confirm that these cells are enriched for OCPs, we compared their surface expression of CD11b and Gr-1 proteins, cell surface markers for OCPs [20], with primary bone marrow mononuclear cells isolated from the same mice.

**Figure 1:**
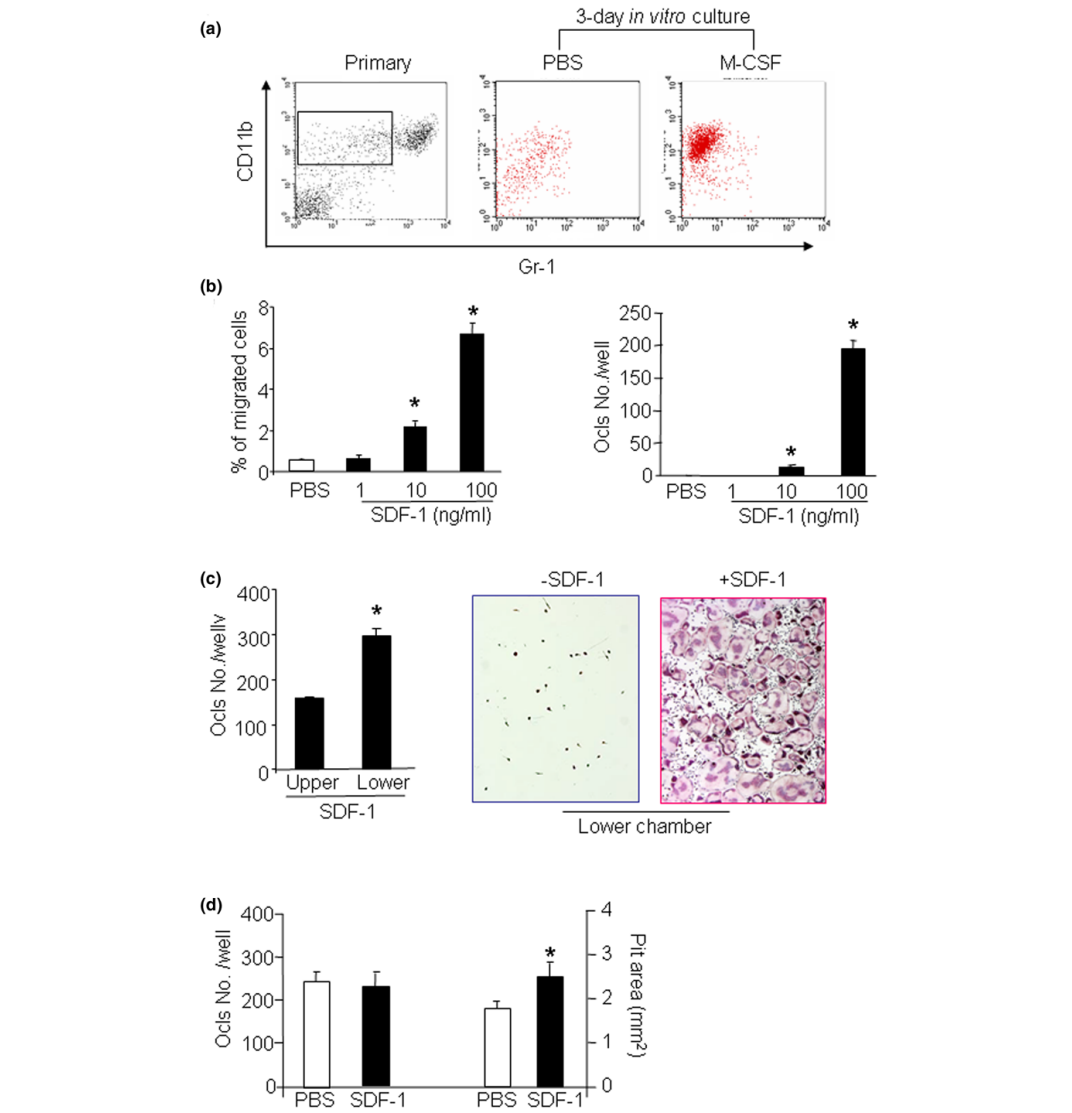
**Effect of stromal cell-derived factor 1 on osteoclast precursor migration and differentiation**. **(a) **Wild-type bone marrow cells were cultured with PBS or macrophage colony-stimulating factor (M-CSF) for 3 days to generate osteoclast precursors (OCPs). Cells were stained with allophycocyanin-labeled anti-CD11b and Phycoerythrin-labeled anti-Gr-1 antibodies and were subjected to fluorescence-activated cell sorting analysis. Distributions of CD11b^+ ^and Gr-1^-/lo ^cells are shown. Rectangle (CD11b^+^/Gr-1^-/lo ^fraction), the majority of cells with osteoclast forming potency. **(b) **Wild-type OCPs were labeled with calcein AM and were seeded in the upper chamber of a transwell dish, and various amounts of stromal cell-derived factor 1 (SDF-1) were added to the lower chamber. Percentage of migrated cells in the lower chamber determined by calcein intensity (left panel). Cells that migrated to the lower chamber were cultured with M-CSF and RANKL to form osteoclasts. Numbers of tartrate-resistant acid phosphatase-positive (TRAP^+^) cells per well was assessed (right panel). **(c) **OCPs were seeded in the upper chamber of a transwell with or without 100 ng/ml SDF-1 in the lower chamber for 3 hours. Nonmigrated cells from the upper chamber and migrated cells from the lower chamber were cultured with M-CSF and RANKL to form osteoclasts. TRAP staining was formed. Bar graphs, numbers of TRAP^+ ^cells/well (left panel). Representative pictures show TRAP-stained osteoclasts formed from the lower chambers with or without SDF-1 (×10). **(d) **OCPs were cultured with M-CSF and RANKL plus SDF-1 (200 ng/ml) on bone slices for 9 days. Numbers of osteoclasts and resorption pits per slice were counted. Data are the mean ± standard error of the mean of four wells. Experiments were repeated three times with similar results. *P < 0.05 versus samples from PBS-treated cells.

As we reported previously [20], more than 10% of primary bone marrow cells are CD11b^+^/Gr-1^-/lo^. After 3 days of culture with M-CSF, more than 90% of adherent bone marrow cells become CD11b^+^/Gr-1^-/lo ^– cells indicating enrichment of OCPs (Figure 1a). We then demonstrated that these OCPs migrated to SDF-1 gradients in a dose-dependent manner (Figure 1b, left panel). The maximum chemotaxic response was observed at 100 ng/ml SDF-1 and did not increase further with up to 250 ng/ml SDF-1 (data not shown). No migration occurred when SDF-1 was included in both the upper and lower chambers.

To confirm their osteoclast forming potency, we cultured the OCPs that had migrated to the SDF-1 gradient with M-CSF and RANKL, and demonstrated that they formed tartrate-resistant acid phosphatase-positive osteoclasts (Figure 1b, right panel). We also cultured nonmigrated OCPs from the upper chamber with M-CSF and RANKL, and compared their osteoclast forming potency with those that have migrated to SDF-1 gradient (100 ng/ml) in the lower chamber. Both nonmigrated and migrated OCPs can give rise to mature osteoclasts, but the cells from the lower chamber formed more osteoclasts (Figure 1c, left panel). In contrast, cells that were freely migrated to the lower chamber without a SDF-1 gradient did not form osteoclasts under the same condition (Figure 1c, right panel). These findings suggest that both nonmigrated and SDF-1 migrated cells can differentiate into osteoclasts but that CXCR4-positive cells have more osteoclast forming potency.

To study the effect of SDF-1 on OCP differentiation and activation, OCPs were cultured with M-CSF and RANKL in the presence or absence of SDF-1 (200 ng/ml) for 9 days on bone slices. SDF-1 did not affect osteoclast numbers, but slightly increased osteoclast resorptive activity (Figure 1d). SDF-1 had no effect on OCP production of TNF. In contrast, RANKL significantly increased TNF expression under the same culture conditions (fold induction of TNF over PBS: RANKL, 11.6 ± 0.9 versus SDF-1, 0.7 ± 0.1). The major role of SDF-1 in the regulation OCPs therefore appears to affect their mobilization through chemotaxis.

### TNF reduces SDF-1 production by bone marrow stromal cells

**Figure 2:**
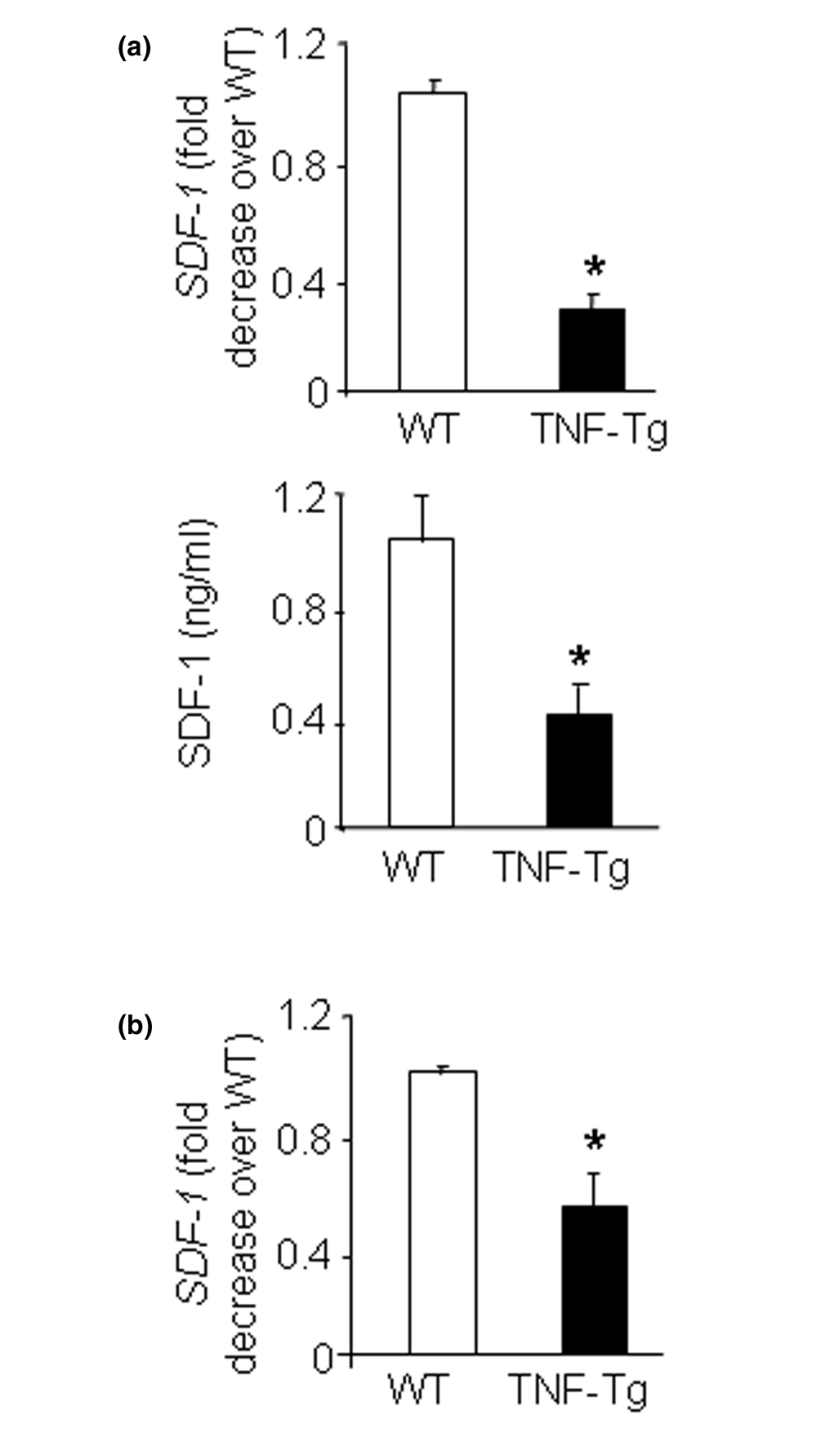
**Decreased stromal cell-derived factor-1 expression in bone marrow stromal cells and bones of TNF-transgenic mice**. **(a) **Bone marrow stromal cells from 6-month-old TNF-transgenic (TNF-Tg) mice and wild-type (WT) littermates were cultured in α-modified essential medium plus 20% fetal bovine serum for 7 days. The stromal cell-derived factor 1 (SDF-1) protein concentration in the conditioned medium was assessed by ELISA (upper panel). Expression levels of SDF-1 mRNA were determined by real-time RT-PCR (lower panel). Fold changes were calculated using the value from WT mice as 1. **(b) **Long bones from the above mice were harvested and subjected to RNA extraction. Expression of SDF-1 was measured by real-time RT-PCR. Data are the mean ± standard error of the mean of three loadings. The same results were obtained from three pairs of TNF-Tg mice and WT littermates. *P < 0.05 versus samples from WT littermates.

Since an SDF-1 gradient determines the direction of mobilization of hematopoietic stem cells and progenitors [6], we examined whether SDF-1 levels are decreased in bone marrow stromal cells and long bone samples from TNF-Tg mice to account for the increased OCP mobilization from their bone marrow to their peripheral blood. SDF-1 mRNA and protein levels were significantly reduced in the bone marrow stromal cells (Figure 2a) and in the long bones from TNF-Tg mice compared with wild-type littermates (Figure 2b).

**Figure 3:**
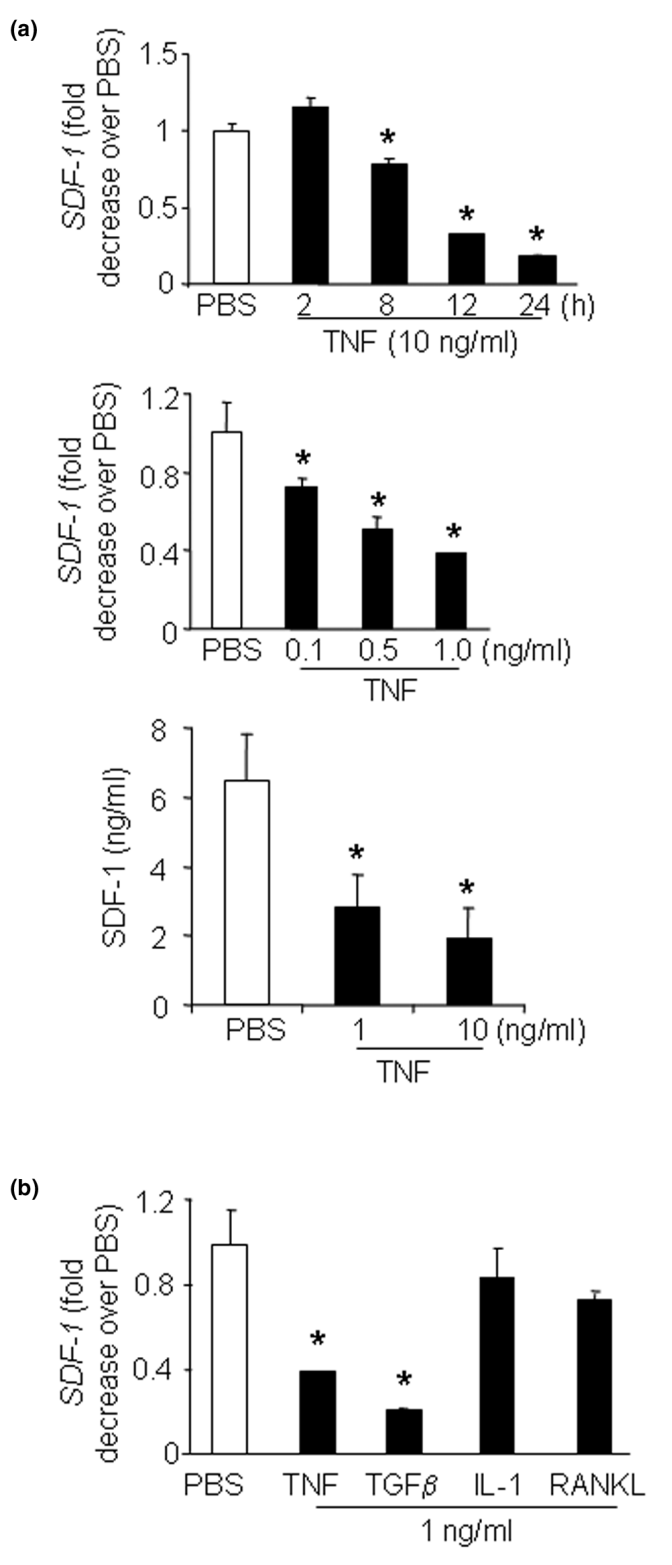
**TNF inhibits stromal cell-derived factor 1 expression by ST2 stromal cells**. ST2 cells, a bone marrow stromal cell line, were treated with TNF or osteoclastogenic cytokines, and expression of stromal cell-derived factor 1 (SDF-1) mRNA was determined by real-time RT-PCR. **(a) **Data from the cells treated with TNF (10 ng/ml) for various time points (upper panel) or different amounts of TNF for 24 hours (middle panel). Changes in SDF-1 protein levels in the conditioned medium were determined by ELISA 24 hours after TNF treatment (lower panel). **(b) **Data from the cells treated with osteoclastogenic cytokines for 24 hours. Data are the mean ± standard error of the mean of three loadings. Data are representative of two independent experiments. *P < 0.05 compared with PBS-treated cells. TGFβ, transforming growth factor beta.

To examine whether TNF directly affects SDF-1 production, we treated ST2 cells – a bone marrow-derived cell line – with TNF, and found that SDF-1 expression decreased within 8 hours and with a relatively low dose of TNF (0.1 ng/ml) (Figure 3a). TNF-reduced SDF-1 production was also confirmed at protein levels (Figure 3a). Other osteoclastogenic cytokines, including IL-1 and RANKL, had no effect on SDF-1 expression (Figure 3b), while TGFβ significantly reduced SDF-1 mRNA expression, as reported previously [16].

**Figure 4:**
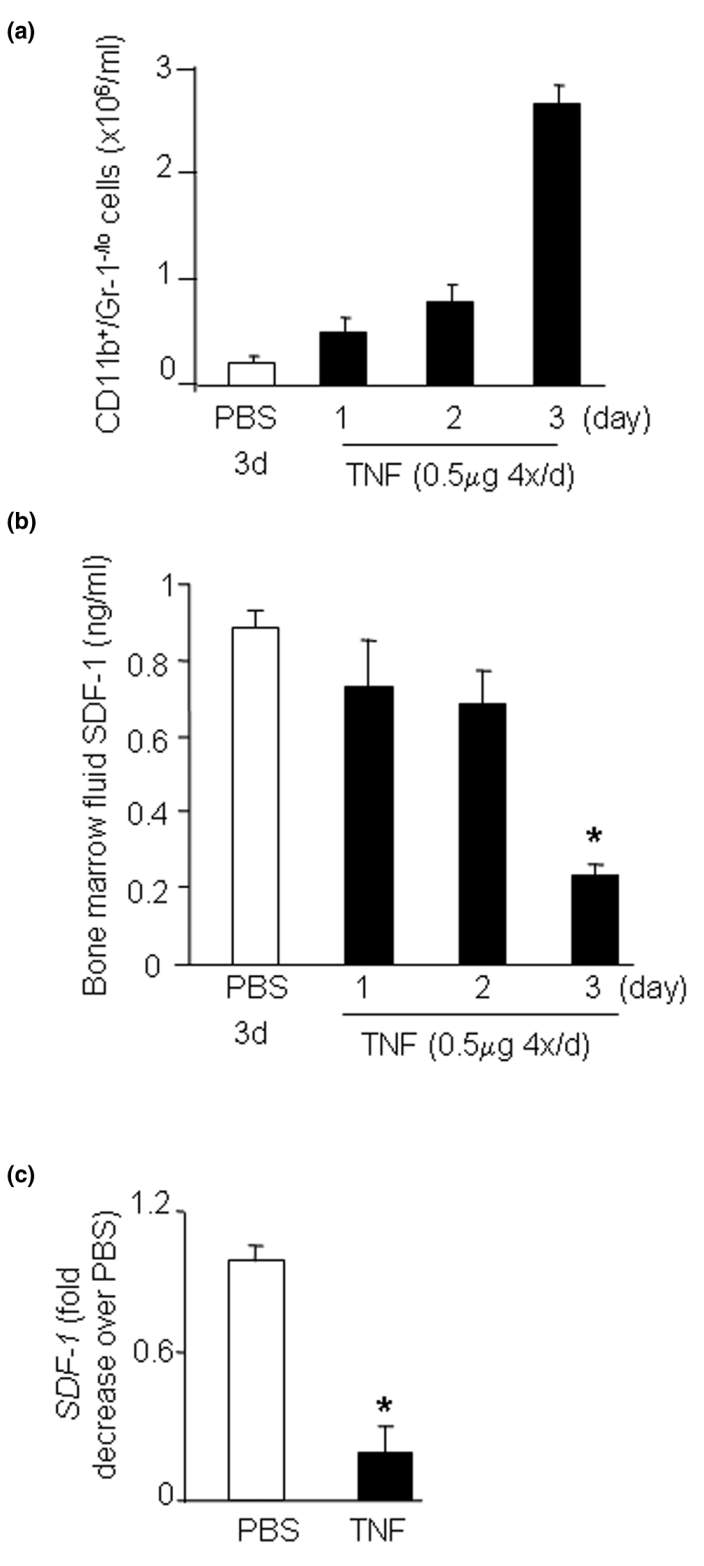
**TNF injection decreases bone marrow stromal cell-derived factor-1 levels and increases blood osteoclast precursor frequency**. Wild-type mice (3/group) were given subcutaneous injections of murine TNF (0.5 μg/injection, 4 times/day) or PBS for 3 days and were sacrificed 2 hours after the last injection on the fourth day. **(a) **The circulating CD11b^+^/Gr-1^-/lo ^osteoclast precursor frequency was determined by fluorescence-activated cell sorting analysis. **(b) **Stromal cell-derived factor 1 (SDF-1) levels in the bone marrow were measured by ELISA. **(c) **Expression of SDF-1 mRNA in bone marrow was determined by real-time RT-PCR. Data are the mean ± standard error of the mean of three pairs of mice receiving TNF or PBS injection. *P < 0.05 versus blood or PBS-treated mice.

**Figure 5:**
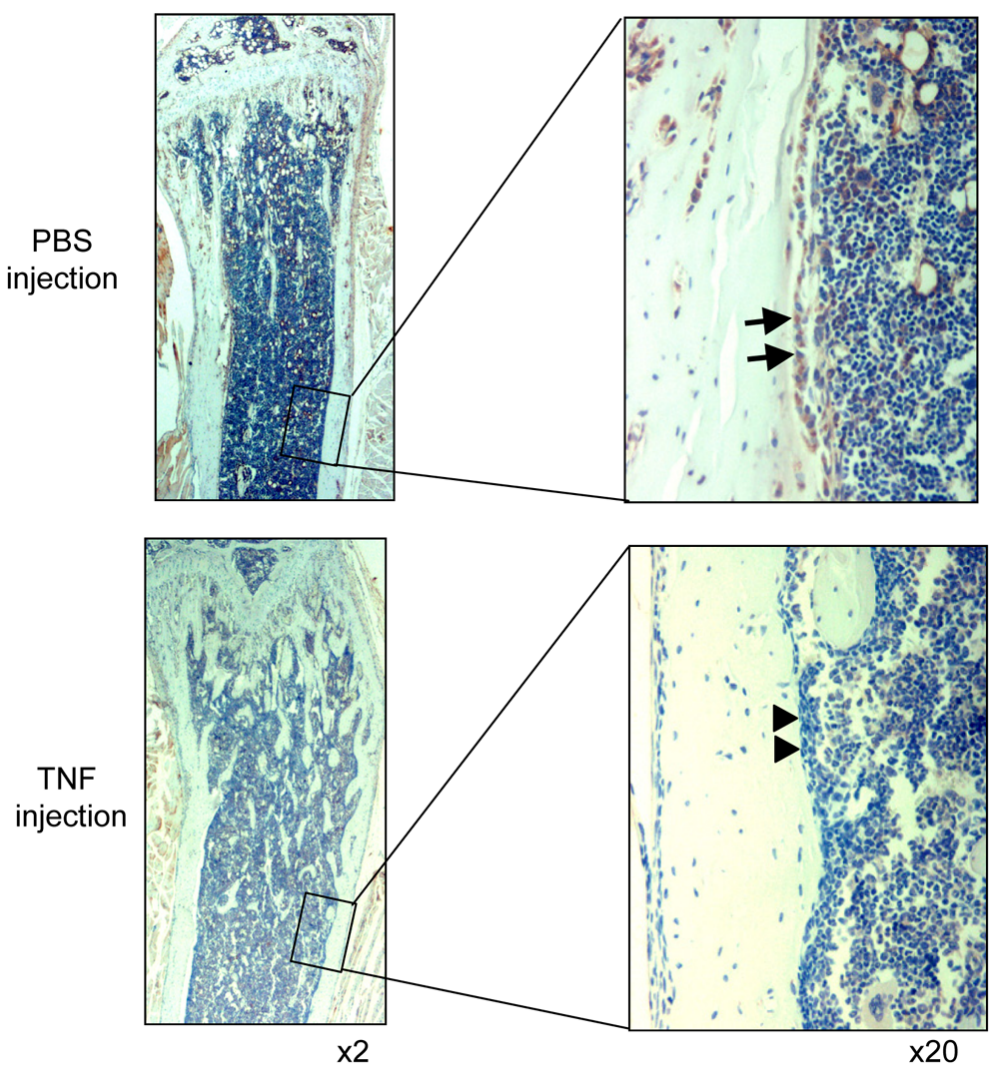
**TNF injection decreases stromal cell-derived factor 1 expression by bone marrow stromal cells**. Expression of stromal cell-derived factor 1 (SDF-1) protein by bone marrow cells was examined by immunostaining with anti-SDF-1 antibody. Left panels: pictures taken at power 2 to show the overall view of bone architecture. Right panels: pictures are the insert taken at power 20 to show SDF-1-positive bone marrow osteoblasts (upper panel arrows) in mice receiving PBS injection and to show SDF-1-negative cells (lower panel arrows) in TNF-injected mice. Photographs are representatives of one pair of TNF-injected or PBS-injected mice from three pairs of animals.

To determine whether the reduction in bone marrow expression of SDF-1 induced by TNF leads to OCP mobilization, we treated wild-type mice with TNF using a subcutaneous injection protocol shown previously to increase the OCP frequency in the blood [4,20]. As expected, TNF increased the blood OCP numbers (Figure 4a). It also decreased SDF-1 protein levels in bone marrow extracts (Figure 4b). The concentration of SDF-1 in bone marrow was thus reduced significantly. Consistent with the SDF-1 protein data, SDF-1 mRNA expression was significantly decreased in the bone marrow cells of TNF-treated mice (Figure 4c). As a control, TGFβ mRNA levels did not change in the same samples (data not shown). Immunostaining with an anti-SDF-1 antibody showed that SDF-1 is strongly expressed by osteoblasts on endosteal and trabecular bone surfaces of murine long bones (Figure 5, arrows). TNF treatment was associated with loss of SDF-1-positive staining of these cells without affecting the cell morphology (Figure 5, arrow heads), indicating that TNF inhibits SDF-1 production by marrow osteoblasts.

### TNF does not affect CXCR4 expression by osteoclast precursors

**Figure 6:**
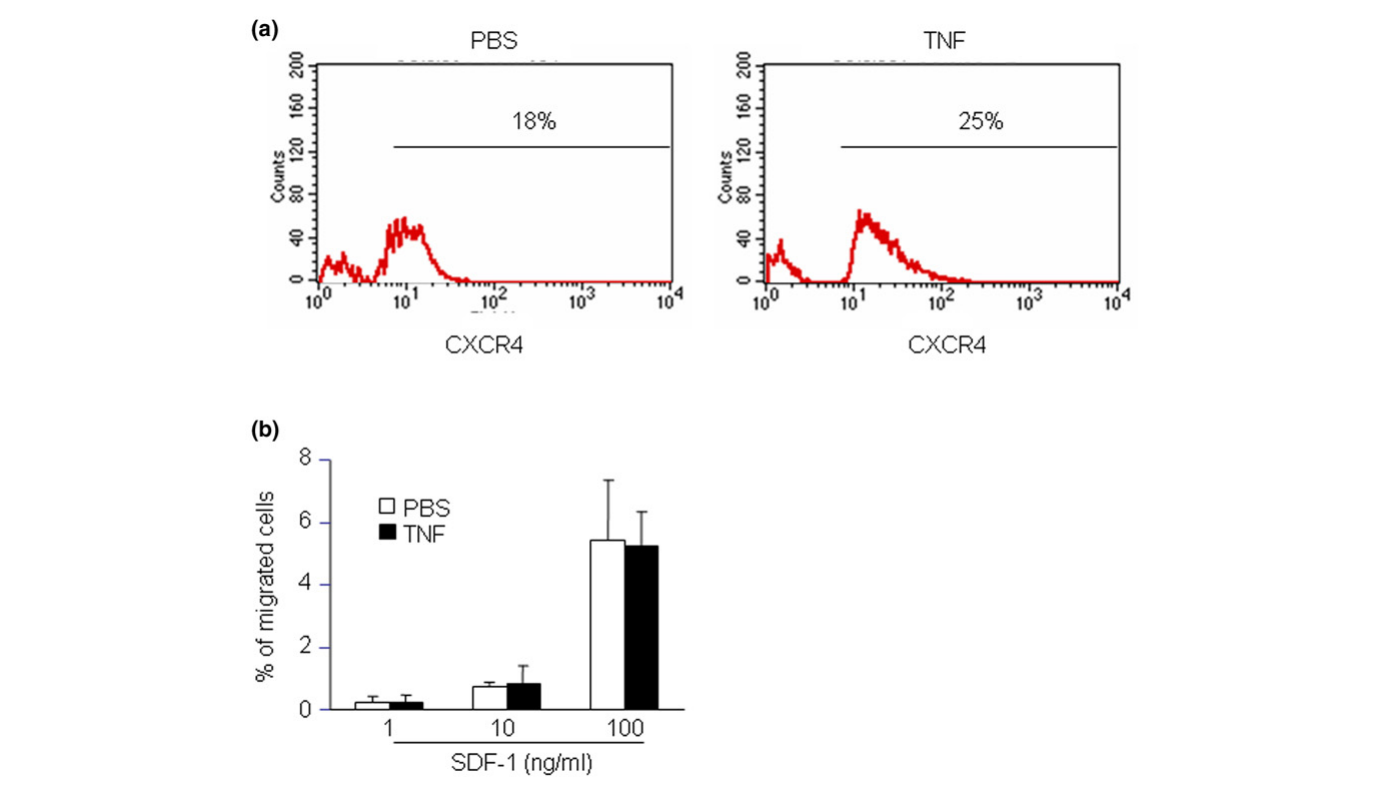
**TNF does not alter CXCR4 expression on osteoclast precursors**. Wild-type bone marrow cells were cultured with macrophage colony-stimulating factor for 3 days and then treated with PBS or TNF (10 ng/ml) for 24 hours. **(a) **Cells were harvested and stained with Phycoerythrin-labeled anti-c-fms and FITC-labeled anti-CXCR4 antibodies and were subjected to fluorescence-activated cell sorting analysis. c-Fms-positive cells were gated and the cell surface expression level of CXCR4 is shown in the histogram. **(b) **Cells were subjected to a transwell assay where various amounts of stromal cell-derived factor 1 (SDF-1) were added in the lower chamber. After 3 hours of incubation, migrated cells in lower chamber were harvested and measured in a microplate spectrofluorometer. Percentage of migrated cells was determined as in Figure 1. Data are representative of two independent experiments.

CXCR4 is the sole receptor for SDF-1, and CXCR4 knockout mice die during embryonic development due to impaired cell homing in the bone marrow [6]. To determine whether the number of CXCR4^+ ^cells is altered in TNF-Tg mice, the percentage of bone marrow CXCR4^+^/CD11b^+^/Gr-1^-/lo ^OCPs was examined by FACS analysis. No difference was observed in the percentage of CXCR4^+^/CD11b^+^/Gr-1^-/lo ^cells between TNF-Tg mice and wild-type littermates (data not shown). TNF pretreatment of wild-type OCPs *in vitro *had no effect on CXCR4 expression on the cell surface (Figure 6a), and OCP migration to SDF-1 gradients was similar between PBS-pretreated and TNF-pretreated cells (Figure 6b). TNF therefore does not appear to influence the expression of CXCR4 by OCPs.

**Figure 7:**
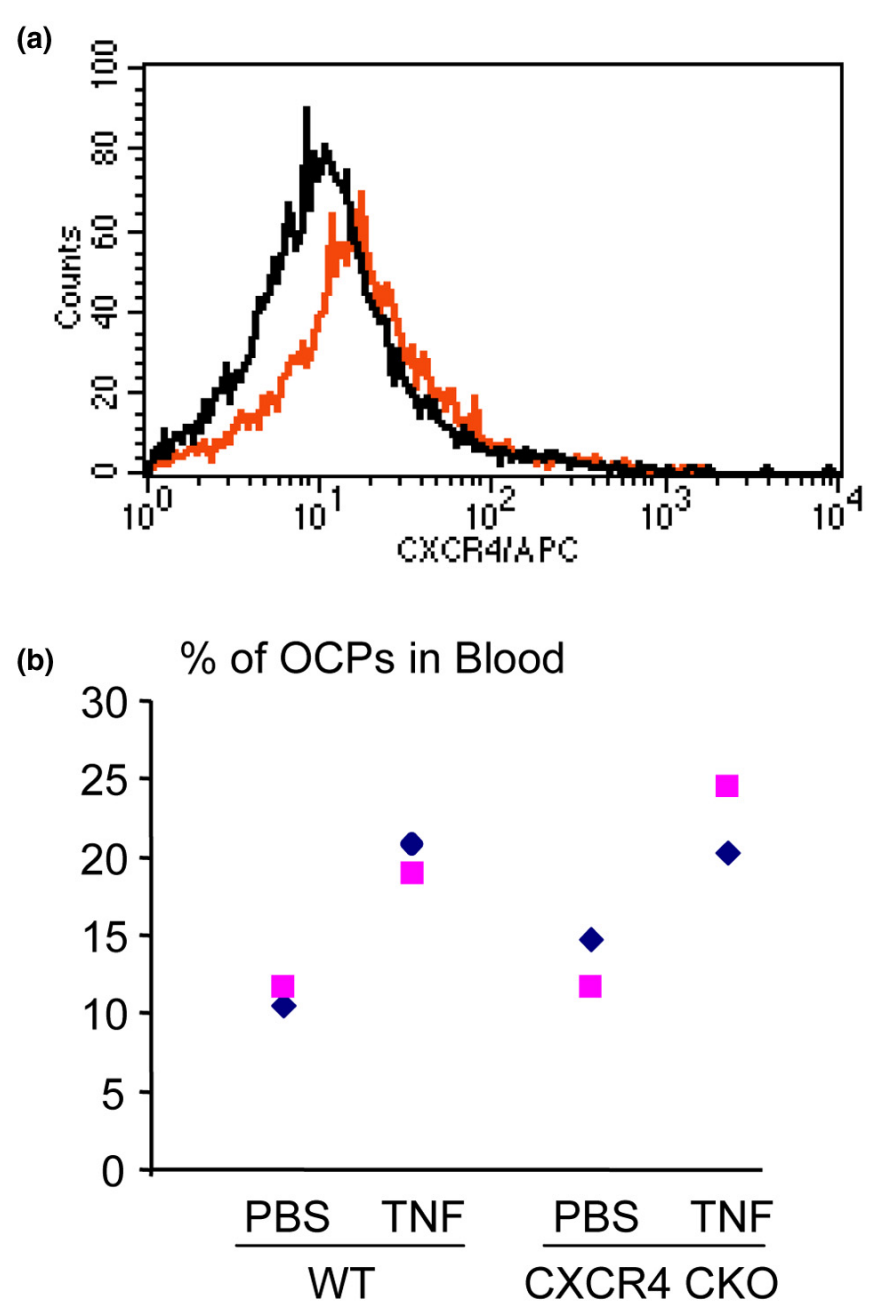
**No changes in TNF-induced osteoclast precursor release from *Cxcr4 f/f*/*CD11b*^+^*/Cre *conditional knockout mice bone marrow**. **(a) **Bone marrow cells from 2-month-old CXCR4 CKO and control mice were double stained with FITC-anti-CD11b and allophycocyanin (APC)–anti-CXCR4 antibodies, and CD11b^+ ^cells were gated. Intensity of CXCR4^+ ^stained CD11b^+ ^cells shown. Black line, CXCR4 CKO cells; red line, control cells. **(b) **Murine TNF (0.5 μg/injection, 4 times/day × 3 days, intraperitoneally) or PBS was injected into 2-month-old CXCR4 CKO mice and control mice (n = 2 per group). Two hours after the last TNF injection, the peripheral blood was harvested and double stained with APC–anti-CD11b and Phycoerythrin-anti-Gr1 antibodies. The percentage of CD11b^+^/Gr-1^-/lo ^osteoclast precursors (OCPs) were determined. Values from individual mice are plotted. bp, base pairs; WT, wild-type.

To determine whether specific deletion of CXCR4 protein in the OCPs affects TNF-induced OCP mobilization, we generated *Cxcr4f/f/CD11b*^+^*/Cre *conditional knockout (CXCR4 CKO) mice. FACS analysis of bone marrow CD11b^+^cells from adult CXCR4 CKO mice indicate that more CD11b^+ ^cells from CXCR4 CKO mice are CXCR4-negative (49% in CXCR4 CKO mice versus 22% in control mice; Figure 7b). We administered TNF (0.5 μg/injection, 4/day for 3 days) to CXCR4 CKO mice and their *Cxcr4f/f/CD11b*^-^*/Cre *control mice, and assessed the blood OCP frequency by FACS analysis. No clear difference in the percentage of CD11b^+^/Gr-1^-/lo ^OCPs between TNF-treated CXCR4 CKO mice and control littermates was observed (Figure 7).

## Discussion

Increased numbers of OCPs have been reported in the peripheral blood of mice in several animal models of arthritis [4,5] and in patients with arthritis [3], but the mechanisms that mediate this increase have not been elucidated. In the present study, we investigated whether the SDF-1/CXCR4 axis is involved in TNF-mediated OCP mobilization because this chemokine system plays an essential role in hematopoietic stem cell and progenitor homing [6]. We found that TNF directly inhibits bone marrow stromal cell production of SDF-1 and reduces SDF-1 levels in the bone marrow, which is accompanied with an increase in the egress of OCPs from the marrow. Decreased SDF-1 production by bone marrow stromal cells in response to TNF overexpression may therefore be one of the mechanisms mediating release of OCPs to the peripheral blood in mice with TNF-induced arthritis or in patients with inflammatory arthritis.

SDF-1-regulated cell mobilization is determined by local SDF-1 gradients and/or CXCR4 expression on target cells. Although alternation of either of these could lead to impaired cell mobilization and homing, external factor regulation of SDF-1 expression levels appears to be the major mechanism. For example, hypoxia [15], DNA damage [14], proteases [21] and cytokines – including TGFβ [16] and G-CSF [17] – all reduce SDF-1 levels and stimulate hematopoietic stem cell release from bone marrow. Regulation of CXCR4 expression by external factors has been studied less and the results have been inconsistent. This inconsistency may be related to small numbers of CXCR4-expressing cells and low expression levels by these cells, making it difficult to reliably detect a change in the number of CXCR4-positive cells.

Our findings that TNF significantly decreases SDF-1 levels but has little effect on OCP CXCR4 expression suggest that, like most hematopoietic cell mobilizers, TNF also promotes OCP mobilization through regulation of SDF-1 rather than through CXCR4 expression. TNF-mediated OCP mobilization, however, is different from stem cell and precursor mobilization induced by SDF-1, G-CSF or other agents because TNF also has a strong stimulatory effect on OCP generation. This represents a unique pathologic situation in chronic inflammatory arthritis, in that the entire process of generation of OCPs and their egress from the bone marrow is accelerated in response to TNF. This situation leads to increased numbers of OCPs in both bone marrow and blood, whereas SDF-1 or G-CSF administration triggers a rapid release of cells from the bone marrow – and the total bone marrow cell number is consequently reduced.

We do not currently know the molecular mechanisms by which TNF inhibits SDF-1 production. SDF-1 is regulated at both transcriptional and post-translational levels [16,21]. We found that TNF induced massive apoptosis of ST2 cells when a transcription or translation inhibitor was used with TNF (data not shown). In these circumstances it is therefore difficult to investigate the mechanism of action for TNF. Protease degradation is one of the major mechanisms to reduce SDF-1 protein levels [21], and protease release from neutrophils and other myeloid cells can be stimulated by TNF. However TNF may also inhibit SDF-1 expression at the RNA level within 8 hours of treatment as shown by our data (Figure 3a).

TGFβ at concentrations as low as 0.01 ng/ml decreases SDF-1 mRNA expression in stromal cells [16], implying that a small change in TGFβ could alter SDF-1 concentrations. We found that TNF increases TGFβ mRNA expression in ST2 cells. TNF administration to wild-type mice had no effect on TGFβ expression, however, although it significantly decreased SDF-1 expression in bone marrow stromal cells. Therefore it is unlikely that TGFβ mediates TNF-induced bone marrow SDF-1 downregulation *in vivo*. G-CSF is another cytokine that downregulates SDF-1 mRNA expression in osteoblasts [17]. TNF did not increase G-CSF in ST2 cells (data not shown), however, suggesting that the reduction in SDF-1 induced by TNF *in vitro *is not mediated by G-CSF. Furthermore, the SDF-1 promoter does not contain binding sites typically present in the other CXC chemokine promoters, especially for NF-κB, interferon regulatory factor recognition elements or NF-IL6, which are associated with transcriptional activation in response to proinflammatory extracellular signals, such as TNF, IL-6 or interferons [22]. These data suggest that studying SDF-1 regulation may be more complicated than studying other CXC chemokines.

The present study did not provide a direct association between TNF-reduced SDF-1 production and OCP mobilization *in vivo*. We have attempted to answer this question using mice with CXCR4 specifically deleted in OCPs by generating CXCR4 CKO mice via crossing CXCR4 floxed mice [18] with CD11b-Cre mice [19]. We injected TNF to these CXCR4 conditional mice to determine whether TNF-induced increased OCP release is altered when CXCR4 expression has theoretically been deleted in CD11b-expressing OCPs. Unfortunately, we found that only about 50% of bone marrow CD11b^+ ^cells have no CXCR4 surface expression in these CXCR4 CKO mice (Figure 7a), suggesting a low excision frequency of the Cre recombinase in our system. With this leaky system, the blood OCP frequency was similar between CXCR4 CKO mice and wild-type mice (Figure 7b). Our results suggest that CD11b-Cre mice appear not a good system to delete the gene encoding *cxcr4 *in bone marrow CD11b-positive cells.

The importance of TNF-mediated reduction in SDF-1 production in increased OCP mobilization *in vivo *needs to be further confirmed using a model where SDF-1 concentration in the bone marrow is maintained in the presence of TNF. Since rheumatoid arthritis and other forms of inflammatory bone disorders are chronic diseases, however, multiple factors may contribute to promote OCP release from the bone marrow. For example, we have demonstrated that TNF-stimulated OCP formation could increase the OCP pool in bone marrow and push cell egression [20]. Kindle and colleagues reported that TNF activates endothelial cells and increases the attachment of OCPs to vascular endothelium *in vitro. *They speculated that this could increase the ability of OCPs to enter the bloodstream [23]. It has been reported recently that RANKL-stimulated osteoclastogenesis promotes the mobilization of hematopoietic progenitor cells by cleaving SDF-1 through bone-resorbing proteinase, cathepsin K [24]. TNF stimulates osteoclastogenesis synergistically with RANKL [25], and this mechanism may also apply to TNF-induced OCP mobilization. The regulation of OCP mobilization is therefore a complicated process, and decreased SDF-1 expression by bone marrow stromal cells may represent another important mechanism.

## Conclusion

Our findings demonstrate that TNF directly inhibits bone marrow stromal cells to produce SDF-1, which is associated with increased release of OCPs from the bone marrow. The SDF1/CXCR4 axis therefore may not only control hematopoietic cell homing, but may also contribute to the accelerated OCP mobilization in inflammatory arthritis where systemic TNF levels are elevated.

## Abbreviations

CKO = conditional knockout; ELISA = enzyme-linked immunosorbent assay; FACS = fluorescence-activated cell sorting; FITC = fluorescein isothiocyanate; G-CSF = granulocyte colony-stimulating factor; IL = interleukin; M-CSF = macrophage colony-stimulating factor; NF = nuclear factor; OCPs = osteoclast precursors; PBS = phosphate-buffered saline PCR = polymerase chain reaction; RANKL = receptor activator of nuclear factor-B ligand; RT = reverse transcriptase; SDF-1 = stromal cell-derived factor 1; Tg = transgenic; TGFβ = transforming growth factor beta; TNF = tumor necrosis factor.

## Competing interests

The authors declare that they have no competing interests.

## Authors' contributions

LX had full access to all data in the study and takes responsibility for the integrity of the data and the accuracy of the data analysis. Study design was by LX, QZ, EMS, and BFB. LX, QZ, and RG were responsible for acquisition of data. Analysis and interpretation of data were performed by LX, QZ, RG, EMS, and BFB. LX, EMS, BFB, and QZ prepared the manuscript. Statistical analysis was performed by QZ and RG.
